# Dysesthesia associated with GLP-1 agonist therapies: data-mining analysis and literature review

**DOI:** 10.1007/s00228-026-04079-7

**Published:** 2026-05-22

**Authors:** Marie-Laure Laroche, Hélène Géniaux, Manon Jardou

**Affiliations:** 1 Regional Center of Pharmacovigilance, Department of Pharmacology, Toxicology and Pharmacovigilance, Limoges’ University Hospital, Limoges, France; 2 Faculty of Medicine, Department of Pharmacology, University of Limoges, 2 avenue Dr Marcland, Limoges, 87000 LIMOGES Cedex France

**Keywords:** GLP-1 receptor agonists, Dysesthesia, Pharmacovigilance

## Abstract

**Purpose:**

An increasing number of anecdotal reports on social media platforms and medical blogs describe dysesthesia, particularly burning skin sensations, in association with glucagon-like peptide-1 receptor (GLP-1R) agonists. We performed a pharmacovigilance data-mining analysis to characterise cases of dysesthesia related to GLP-1R.

**Methods:**

We conducted a disproportionality analysis using VigiBase data on GLP-1R (Anatomical Therapeutic Chemical classification: ATC code A10BJ) and tirzepatide, focusing on the HLT (High Level Term) “Paraesthesia and dysesthesia”, with the Information Component (IC). Additionally, we reviewed all narratives of dysesthesia cases in the French Pharmacovigilance database to extract clinical and pharmacological characteristics. A literature review complemented this analysis.

**Results:**

Exenatide was significantly associated with hypoesthesia or oral paraesthesia, semaglutide and tirzepatide with hyperaesthesia, and semaglutide with dysesthesia and burning sensation. Dysesthesia appears to be dose-dependent, occurring more frequently at higher doses and with more potent GLP-1R, whether used for weight management or type 2 diabetes. Discontinuation was often performed, followed by spontaneous favourable outcomes, and cases of rechallenge were observed. Skin burning sensations represent a distinctive form of dysesthesia.

**Conclusion:**

Pharmacovigilance quantitative and qualitative data strengthens evidence for dysesthesias associated with GLP-1R agonists already observed in clinical trials of semaglutide, tirzepatide, and retatrutide.

**Supplementary Information:**

The online version contains supplementary material available at 10.1007/s00228-026-04079-7.

## Introduction

Glucagon-like peptide-1 (GLP-1) agonist therapies are used for type 2 diabetes treatment as well as weight management. They currently comprise three categories: single agonists targeting the glucagon-like peptide-1 receptor (GLP-1R) (albiglutide, exenatide, dulaglutide, liraglutide, lixisenatide, semaglutide, and orforglipron under-development), dual agonists targeting GLP-1R and the glucose-dependent insulinotropic polypeptide receptor (GIPR) (tirzepatide) and triple agonists simultaneously targeting the GLP-1R, GIPR and the glucagon receptor (GCGR) (retatrutide, under-development). These multi-agonists act on multiple key metabolic pathways simultaneously, providing more comprehensive regulation of glucose homeostasis, body weight, and overall metabolic health [1].

The GLP-1R is expressed not only in the pancreas but also in numerous other organs and tissues such as gastrointestinal tract, cardiovascular system, and central nervous system [2]. Despite their broad range of targets, GLP-1R agonists are associated with various adverse effects. Among them, gastrointestinal adverse event are commonly reported [3]. Moreover, emerging data suggest that GLP-1R agonists might be associated with neurological adverse event [4]. Several reports have suggested that GLP-1R agonists may exert neuroprotective properties in both central and peripheral nervous system (PNS) [5, 6]. However, an increasing number of anecdotal reports from patients on social media platforms and medical blogs have described dysesthesia, particularly burning skin sensations, in association with GLP-1 receptor agonists. Dysesthesia refers to abnormal sensations in the skin that may manifest in various forms (anaesthesia, hypoesthesia, hyperesthesia, paraesthesia, burning sensation…). It is considered a clinical sign of peripheral neuropathy, reflecting damage to the PNS [7]. The most common classes of drug known to induce peripherical neuropathy include chemotherapies, cardiovascular agents, antibiotics, and antiretrovirals [7]. Symptoms typically develop weeks to months after treatment initiation and may vary in intensity and distribution.

The current medical literature provides limited information regarding dysesthesia associated with GLP-1R agonists. The aim of the paper is to perform a data-mining analysis of pharmacovigilance databases, completed by a review of French narratives cases and a literature review, to document and characterise cases of dysesthesia related to GLP-1 analogues therapies.

## Methods

### Data-mining in the international pharmacovigilance database (VigiBase)

This step allows for the detection of drug-adverse event combinations that are reported more frequently than expected, thereby suggesting a potential safety signal. The study was conducted in accordance with the READUS-PV guideline (“The Reporting of a disproportionality analysis for drug Safety signal detection using spontaneously reported adverse events in Pharmacovigilance (READUS-PV)”, https://readus-statement.org) [8].

Data were extracted from the Uppsala Monitoring Centre (UMC) global pharmacovigilance database VigiBase. VigiBase holds over 33 million anonymised spontaneous individual case safety reports (ICSRs) of suspected adverse events related to medicinal products (as of January 2025), submitted by 130 member countries since 1967 [9]. Its primary purpose is the detection of potential medicine safety signals. Adverse events in VigiBase are coded using the Medical Dictionary for Regulatory Activities (MedDRA– Medical Dictionary for Regulatory Activities, version 28.1) at the level of Preferred Terms (PTs). Each PT represents a single medical concept and is hierarchically linked to broader High Level Terms (HLTs), HighLevel Group Terms (HLGTs), and System Organ Classes (SOCs), in accordance with the MedDRA Hierarchy. Cases of interest were identified under the HLT “Paraesthesia and dysesthesia”, after excluding cases reported as administration site reaction. All reports involving GLP-1R agonists (ATC code A10BJ) and tirzepatide, recorded in Vigibase up until the 25th of October 2025 were identified.

A descriptive analysis was carried out and results were presented as frequencies and percentages. A disproportionality analysis using VigiBase data was also performed, based on the information component (IC). The IC is a bayesian indicator developed by the Uppsala Monitoring Centre to measure disproportionate reporting [10, 11]. This method compares the proportion of a given adverse event associated with a specific drug to the proportion of the same adverse event for all other drugs in VigiBase. The IC was selected for its conservative properties, minimizing the likehood of false-positive signals and thereby strengthening the robustness of the results. IC_025_ represents the lower bound of the 95% credibility interval for the IC estimate. A positive IC value, that usually requires that the lower 95% CI of the IC exceed zero, indicates that a specific drug-adverse event combination is reported more frequently than expected, suggesting a potential safety signal. A positive IC_025_ value (IC_025_ > 0) is the threshold deemed significant and used in statistical signal detection [10, 11]. Thus, an IC_025_ > 0 indicates a statistically significant reporting association between a drug and the adverse event.

### Clinical description of case reports (French Pharmacovigilance Database)

A safety signal requires comprehensive investigation, including a detailed review of individual case reports or case series within a pharmacovigilance database or the published literature. The case narrative contains essential information for causality assessment that is not always fully captured in the structured data fields, such as the time course of events, differential diagnoses considered, or dose-dependent effect. A narrative-based analysis remains crucial to ensure the robustness and reliability of signal evaluation. Because cases in the French Pharmacovigilance Database (FPVD) contain physician- or pharmacist-validated narratives, we reviewed all narratives of dysesthesia cases associated with GLP-1R agonists in this database, applying the same criteria as those used in the VigiBase query. We extracted the clinical characteristics of dysesthesia, the implicated products, differential diagnoses, dose and time to onset, dechallenge and rechallenge informations.

## Results

### Description of cases in international pharmacovigilance database (VigiBase)

Out of a total of 426,945 ICSRs involving GLP-1R agonists, 4,281 (1.0%) were related to dysesthesia, of which 3,405 (79.5%) were classified as non-serious adverse reactions. Among 845 serious cases, 589 (69.7%) were considered medically important condition and 312 (36.9%) resulted in hospitalisation or prolonged it.

Table 1 summarises the main characteristics of ICSRs related to GLP-1R agonists associated with dysesthesia. Most reports originated from the USA (2,762; 64.5%) and were predominantly submitted by consumers/non-health professionals (3,022; 70.6%). Sex distribution were 2,961 women (69.2%) and 1,126 men (26.3%) (194 unspecified). Age stratification suggested that the most affected group was adults aged 45–64 years (2,104 cases, 49.1%). The most frequently reported drugs were semaglutide (1,308 ICSRs, 30.5%), exenatide (1,009 ICSRs, 23.6%) and tirzepatide (808 ICSRs, 18.9%).

**Table 1:** Main characteristics of individual case safety reports (ICSRs) related to GLP-1R agonists associated with dysesthesia collected in VigiBase^®^

Age	Number of cases (*n* = 4,281)	%
0–27 days	4	0.1%
28 days to 23 months	3	0.1%
2–11 years	2	0.0%
12–17 years	6	0.1%
18–44 years	617	14.4%
45–64 years	1,547	36.1%
65–74 years	557	13.0%
≥ 75 years	164	3.8%
Unknown	1,381	32.3%
Sex		
Female	2,961	69.2%
Male	1,126	26.3%
Unknown	194	4.5%
Countries		
United States of America	2,762	64.5%
United Kingdom of Great Britain and Northern Ireland	333	7.8%
Mexico	118	2.8%
Denmark	97	2.3%
Germany	95	2.2%
France	81	1.9%
Reporter qualification		
Physician	637	14.9%
Pharmacist	272	6.4%
Other Health Professional	329	7.7%
Lawyer	4	0.1%
Consumer/Non Health Professional	3,022	70.6%
Unknown	151	3.5%
Products		
Semaglutide	1,308	30.5%
Exenatide	1,009	23.6%
Tirzepatide	808	18.9%
Dulaglutide	601	14.0%
Liraglutide	525	12.3%
Albiglutide	39	0.9%
Lixisenatide	19	0.4%
Seriousness		
Yes	845	19.7%
No	3,405	79.5%
Unknown	31	0.7%
Reaction (PT)*		
Paraesthesia	1,501	35.1%
Hypoesthesia	1,263	29.5%
Burning sensation	761	17.8%
Skin burning sensation	381	8.9%
Paraesthesia oral	257	6.0%
Hypoesthesia oral	234	5.5%
Hyperesthesia	233	5.4%
Dysesthesia	92	2.1%
Formication	43	1.0%

* the most reported term with frequency > 1%

Table 2 summarises the data showing significant positive IC/IC₀₂₅ values: exenatide was associated with hypoesthesia or oral paraesthesia, semaglutide and tirzepatide with hyperaesthesia, and semaglutide with dysesthesia and burning sensation. Appendix 1 provides detailed IC values for all reported GLP-1R agonist–adverse event pairs related to dysesthesia.

**Table 2:** Information component (IC) and IC025 of individual cases of suspected adverse reactions to GLP-1R agonists associated with dysesthesia reported in VigiBase^®^

Drug	Reaction (PT)	Number	IC	IC025*
Exenatide				
	Hypoesthesia oral	89	0.8	0.5
	Paraesthesia oral	79	0.5	0.1
Semaglutide				
	Hyperesthesia	157	2.6	2.4
	Dysesthesia	62	2.1	1.8
	Skin burning sensation. burning sensation	470	0.1	0.0
Tirzepatide				
	Hyperesthesia	52	0.7	0.3

* IC and IC025 > 0 indicate a statistically significant reporting association between a drug and the adverse event

## Clinical description of case reports from the french pharmacovigilance database

A total of 28 ICSR describing dysesthesia associated with GLP-1R agonists were extracted from the French Pharmacovigilance database. After review of all cases, only 15 ICSRs were considered sufficiently informative or not attributable to a local injection-site reaction (Table 3). The most frequently reported drug was semaglutide (7 cases for type 2 diabetes, 3 for weight management), followed by both dulaglutide (4) and liraglutide (1) in type 2 diabetes. The adverse reactions occurred in 9 women (60%) and 6 men (40%). The mean age was 58.7 ± 9.5 years, with BMI values ranging from 25.3 to 45.3 kg/m². A history of neuropathy was reported in 4 patients, including 3 with diabetic neuropathy. No associated drugs known to induce dysesthesia were identified in these cases. The predominant manifestation was burning sensation (8 cases with semaglutide), followed by paraesthesia (5 cases). Burning sensations were mainly localised to the back, abdomen, thighs, and arms, whereas paraesthesia primarily affected the extremities. The dose onset varied widely between patients, occurring during dose escalation. The delay between the first injection and the onset of symptoms ranged from 3 to 93 days. Dysesthesia resolved spontaneously after discontinuation of GLP-1R agonist. When a clinical workup was performed, the findings were unremarkable. In the 3 cases with rechallenge, the same symptoms recurred at the same dose and with a similar dose onset as during the initial episode.

**Table 3:** Description of cases of GLP-1R agonist associated with dysesthesia reported in the French Pharmacovigilance Database

	Sex	Age (years)	BMI (kg/m²)	Neurological history	Reaction (PT)	Location	Dose onset	Dose-dependent	Time to onset (days)	Dechallenge	Rechallenge
Indication Type 2 Diabetes											
Dulaglutide											
	F	57	UNK	No	paraesthesia	breasts	3.0 mg	yes	3	resolved	yes
	F	60	25.4	No	paraesthesia	skull, forehead, ear canal, upper lip	0.75 mg	UNK	UNK	resolved	UNK
	M	53	25.3	UNK	paraesthesia	extremities of the limbs	1.5 mg	UNK	17	resolved	UNK
	F	71	UNK	No	paraesthesia	two hands	1.5 mg	UNK	60	UNK	UNK
Liraglutide											
	F	65	27.2	UNK	hyperesthesia	extremities of the fingers	1.8 mg	UNK	10	resolved	UNK
Semaglutide											
	F	42	45.3	No	burning sensation	back	2.4 mg	yes	UNK	resolved	yes
	F	58	23.8	No	burning sensation	thighs, buttocks, back	0.75 mg	yes	71	UNK	yes
	F	61	30.8	No	burning sensation	back, thighs, hips	1.7 mg	yes	11	resolved	no
	M	50	35.5	UNK	burning sensation	shoulders, arms, back, thighs	1.0 mg	yes	93	UNK	UNK
	M	58	UNK	diabetic neuropathy, fibromyalgia	burning sensation	abdomen, arms, thighs	2.4 mg	yes	UNK	resolved	no
	M	68	28.6	Parkinson disease	burning sensation	arms, forearms, thighs, back	UNK	UNK	27	resolved	no
	M	76	36.1	diabetic neuropathy	hypoesthesia	UNK	UNK	yes	UNK	UNK	UNK
Indication Weight management											
Semaglutide											
	F	61	30.8	No	burning sensation	back, thighs, hips	1.7 mg	yes	11	resolved	no
	M	58	UNK	diabetic neuropathy	burning sensation	abdomen, arms, thighs	2.4 mg	yes	UNK	resolved	no
	F	42	45.3	No	paraesthesia	back	2.4 mg	yes	UNK	resolved	no

*UNK* unknown

## Discussion

Both quantitative (IC) and qualitative pharmacovigilance approaches show consistent clinical patterns. Dysesthesia associated with GLP-1R agonists was more frequently reported with semaglutide, as burning sensations, predominantly in women and in the middle age (45–64 years). This phenomenon can occur in both diabetes and weight management indications. Symptoms appeared during dose escalation, resolved after drug discontinuation, and recurred upon rechallenge, supporting a possible causal relationship.

In the literature, similar situations have also been reported. Three case reports of burning sensation associated with semaglutide and tirzepatide were identified. The first case involved a 56-year-old woman treated with subcutaneous liraglutide up to 3 mg/week for chronic weight management. The dose was gradually titrated from 0.6 mg to 3 mg daily over six months, after which she was switched to semaglutide 2.4 mg weekly [12]. A few weeks after initiating semaglutide, she developed a burning sensation that persisted for approximately six weeks before resolving spontaneously. The second case concerned a 75-year-old man with type 2 diabetes who was initially receiving oral semaglutide 14 mg daily, then later switched to subcutaneous tirzepatide with monthly dose escalation from 2.5 mg up to 15 mg for weight management. He reported a generalized burning sensation affecting his whole body [12]. His medical history included peripheral neuropathy and chronic back pain, treated with pregabalin and duloxetine. Discontinuation of tirzepatide led to complete resolution of symptoms. The patient was subsequently reintroduced to oral semaglutide 14 mg daily, which was well tolerated. He later received subcutaneous semaglutide up to 2 mg weekly without recurrence of dysesthesia. Lastly, the third case involved an 86-year-old man with obesity (BMI 38.5 kg/m²), who was switched from dulaglutide 4.5 mg weekly to semaglutide 2 mg weekly [13]. After the third dose of semaglutide, he experienced a burning sensation over the entire body (arms, legs, chest, abdomen, back, and head), sensitive to light touch and pressure, but without rash, erythema, or visible skin changes. Discontinuation of semaglutide for one month resulted in complete symptom resolution. Two months later, semaglutide was reintroduced at 0.25 mg weekly and slowly titrated with dose increases every 4 to 6 weeks. The patient tolerated semaglutide well at 1 mg weekly and requested to retry 2 mg. Upon re-escalation to 2 mg, he experienced a similar, though less intense, burning sensation. These observations suggest that both oral and subcutaneous formulations of semaglutide and tirzepatide, used for type 2 diabetes and chronic weight management, may induce dysesthesia presenting as a burning sensation. The reaction appears to be dose-dependent.

In clinical trials, semaglutide has been associated to the occurrence of dysesthesia. The OASIS 1 trail evaluated the efficacy of oral semaglutide at a dose of 50 mg daily in adults with obesity or overweight, without type 2 diabetes [14]. Altered skin sensation was reported in 13% of people with semaglutide vs. 1% with placebo, generally mild to moderate in severity, occurred during escalation to higher doses, and resolved without discontinuation of medication. In a scoping review including 15 clinical trials, 6 case reports, and 1 retrospective cohort study, the authors investigate reported dermatologic adverse events associated with both subcutaneous and oral administrations of semaglutide [15]. Oral administration of semaglutide 50 mg weekly was associated with altered skin sensations, such as dysesthesia (1.8%), hyperesthesia (1.2%), neuralgia (0.9%), pain of the skin (2.4%), paraesthesia (2.7%), sensitive skin (2.7%), and a skin burning sensation led to discontinuation of semaglutide (1.8%). Only 0.2% of patients experienced skin burning sensation with subcutaneous semaglutide 2.4 mg/weekly [16]. These cases all occurred in clinical trials conducted for the obesity indication. In a clinical trial assessing the dose-dependent effects of subcutaneous semaglutide at doses up to 16 mg weekly in patients with type 2 diabetes and overweight or obesity, dysesthesia were more frequently reported in the 8 mg (8%) and 16 mg (18%) groups than in the 2 mg (0%) and placebo (2%) groups. In two cases, the treatment was withdrawn, and recovery occurred afterward (a case of sensitive skin in the 8 mg group and a case of paraesthesia in the 16 mg group). In four cases, the participants did not recover within the follow-up period [17].

In the STEP UP and STEP UP T2D trials, participants with obesity without or with type 2 diabetes were randomly assigned to receive once-weekly subcutaneous semaglutide 7.2 mg, 2.4 mg, and placebo [18, 19]. In STEP UP, dysesthesia was observed in 22.9% of the semaglutide 7.2 mg group, 6% of the semaglutide 2.4 mg group, and 0.5% of the placebo group [18]. In STEP UP T2D, dysesthesia was observed in 18.9% of the semaglutide 7.2 mg group, 4.9% of the semaglutide 2.4 mg, and none in placebo [19]. The cases were mild or moderate in severity, and resolved before the end of the trial. However, in the STEP UP trial, one in five people who had dysesthesia in the 7.2 mg group needed a dose reduction, and four people in the 7.2 mg group stopped treatment due to dysesthesia [19].

Regulators are starting to incorporate these observations into product information. For instance, in January 2025, the FDA added “dysesthesia” to the list of post-marketing adverse reactions in the US label for injectable semaglutide for diabetes mellitus. The European Medicines Agency (EMA) had included dysesthesia in the Summary of Product Characteristics (SmPC) for semaglutide and tirzepatide. To date, the SmPCs of other GLP-1R agonists (e.g. exenatide, dulaglutide, liraglutide) do not explicitly mention dysesthesia.

More recently, in a phase 2 trial of the retatrutide, a triple agonist GLP1/GIP/GCG used in weight management, cutaneous hyperesthesia and skin sensitivity adverse events were reported in 7% of the participants who received retatrutide and 1% of those who received placebo, occurring in a dose-dependent manner [20]. These cases were mild to moderate in severity and did not lead to discontinuation of treatment. Finally, in the ATTAIN-1 trial, participants with obesity without diabetes mellitus were randomly assigned to receive orforglipron (an oral small-molecule non-peptide GLP-1R) at one of three doses (6 mg, 12 mg, or 36 mg) or placebo once daily for 72 weeks. Dysesthesia (including allodynia, dysesthesia, burning sensation, hyperesthesia) were also reported with dose-dependent effect (obesity: 0.1% for 6 mg group, 0.1% for 12 mg group, 1.2% for 36 mg group and 0.6% for placebo group [21].

The underlying mechanism remains unknown, although several hypotheses have been suggested. Some researchers have suggested a potential link with the loss of subcutaneous adipose tissue [22]. Nutritional deficiencies, particularly in B vitamins and copper, are known to be associated with sensory neuropathies that are chronic, slowly progressive, and length-dependent [7]. However, these hypotheses are not supported by pharmacovigilance reports, where the time to onset is relatively short, ranging from 3 to 93 days, and a rapid improvement after discontinuing the drug. Moreover, in the STEP UP trial, the increase in dysesthesia cases at the 7.2 mg dose occurred shortly after the direct escalation from 2.4 mg to 7.2 mg, suggesting that this phenomenon is unlikely to be related to weight loss or fat mass reduction [18].

Another hypothesis proposes that GLP-1R agonists may exert indirect effects on nerve function, possibly influencing peripheral nerves through metabolic or vascular pathways [22]. Indeed, sensory neurons express both GLP-1 and GIP receptors [7]. Additional evidence supports a potential physiological role of GIP/GIPR signalling in the peripheral nervous system [5]. Thus, activation of GLP-1 and GIP receptors may alter neuronal signalling and contribute to sensations such as dysesthesia or hyperesthesia [23]. Given that dysesthesia has been observed with both selective GLP-1R agonists and dual or triple GLP-1/GIP or GLP-1/GIP/GCGR agonists (tirzepatide, retatrutide), this hypothesis warrants further investigation.

The main strength of this study lies in its combination of several sources of information on an adverse event that remains poorly recognized by prescribers. The combination of a disproportionality analysis using VigiBase^®^ with a detailed narrative review of cases from the French pharmacovigilance database represents a notable methodological strength. The quantitative component generates robust, hypothesis-generating signals, whereas the qualitative component allows for a fine-grained characterisation of clinical and pharmacological features, including time to onset, dose-dependence, rechallenge cases, and clinical outcomes. Together, these approaches provide a more comprehensive characterisation of dysesthesia associated with GLP-1R agonist therapies. Furthermore, the literature review anchors these findings in clinical trial data, thereby- strengthening their plausibility. Another strength is the clinical relevance of this study, as GLP-1R agonists are increasingly prescribed worldwide for both type 2 diabetes and obesity, making the identification of emerging adverse effects particularly important for public health perspective. Several limitations should be acknowledged. VigiBase^®^ contains data of variable quality depending on the contributing countries, with missing information and diagnostic imprecision. A notoriety bias cannot be excluded. The substantial media and scientific attention surrounding semaglutide and tirzepatide may have led to increased reporting of adverse events for these drugs, which could artificially increase their IC values compared to other molecules. The lack of exposure data is also a bias. Without a precise denominator (prescription volumes per molecule), it not possible to calculate a true incidence rate, and cross-molecule comparisons remain relative. Finally, this is currently no mechanistic explanation for this dose-dependent clinical phenomenon, which have a sufficiently significant clinical impact to lead some patients to discontinue the drug despite its efficacy.

Although the term “dysesthesia” has recently been mentioned in the product information for semaglutide and tirzepatide, it remains broad and non-specific. For future GLP-1R agonists therapies, such as retatrutide, this information should be included in product monographs from the time of marketing authorisation. Burning sensations are rare manifestations of dysesthesia that may raise questions for clinicians. A more precise description of the type of dysesthesia would therefore be more informative for clinical practice. Similarly, dose-dependence and recurrence upon rechallenge at the triggering dose are not currently mentioned in the product monographs. However, these clinical features of GLP-1R agonists-induced dysesthesia are important for clinical practice. There appears to be a discrepancy in the assessment of the appropriate management of dysesthesia occurring after GLP-1R agonists exposure: spontaneous resolution despite treatment continuation in clinical trials versus resolution after treatment discontinuation in real-world practice, with positive rechallenge in some cases. Management should probably be tailored to each patient according to the impact on quality of life. Some patients experience highly disabling dysesthesia requiring treatment discontinuation, even when the drug is otherwise effective.

## Conclusion

Pharmacovigilance quantitative and qualitative data strengthens evidence for dysesthesia associated with GLP-1R agonists, already observed in clinical trials of semaglutide, tirzepatide, and retatrutide. This study helps to characterise this adverse reaction: it appears to be dose-dependent, with higher reporting rates at higher doses and with more potent GLP-1R agonists, whether used for weight management or type 2 diabetes. It tends to recur at the same dose as during the initial episode, and generally shows a favourable outcome upon drug discontinuation or spontaneously. Skin burning sensations represent a distinctive form of dysesthesia, rarely reported with medications, yet they can be particularly distressing for patients. In routine clinical practice, our data suggest that this event frequently leads to GLP-1R agonists discontinuation, which may negatively impact patient treatment adherence. In some cases, the severity of burning sensations may prompt clinicians and patients to reassess the benefit–risk balance of continuing treatment.

## Supplementary Information

Below is the link to the electronic supplementary material.

Supplementary Material 1.

## Data Availability

The datasets generated during and/or analysed during the current study are available from the corresponding author on reasonable request.

## Appendix 1: Information component (IC) and IC025 of individual cases of suspected adverse reactions to GLP-1R agonists associated with dysesthesia reported in VigiBase^®^

**Table:**

Drug	Reaction (PT)	Number	IC	IC025
Albiglutide				
	Burning sensation	12	-0.9	-1.8
	Hypoesthesia	13	-2.0	-2.9
	Paraesthesia	14	-2.1	-3.0
	Skin burning sensation	5	-1.9	-3.4
	hypoesthesia oral	2	-1.2	-3.8
	Paraesthesia oral	1	-2.1	-5.9
Dulaglutide				
	Burning sensation	132	-0.7	-1.0
	hypoesthesia oral	33	-0.6	-1.1
	Pharyngeal paraesthesia	6	0.2	-1.2
	hypoesthesia	253	-1.0	-1.2
	Paraesthesia oral	29	-1.0	-1.5
	Paraesthesia	186	-1.7	-1.9
	Pharyngeal hypoesthesia	2	-0.7	-3.3
	Hyperaesthesia	6	-1.9	-3.3
	Anaesthesia	1	0.3	-3.5
	Formication	5	-2.2	-3.7
	Skin burning sensation	19	-3.3	-4.1
	Burning mouth syndrome	1	-0.3	-4.1
	Dysesthesia	1	-3.2	-7
Exenatide				
	hypoesthesia oral	89	0.8	0.5
	Paraesthesia oral	79	0.5	0.1
	hypoesthesia	376	-0.5	-0.6
	Burning sensation	154	-0.5	-0.8
	Paraesthesia	362	-0.8	-0.9
	Formication	19	-0.4	-1.1
	Hyperaesthesia	13	-0.9	-1.8
	Pharyngeal paraesthesia	3	-0.7	-2.7
	Dysesthesia	4	-1.6	-3.4
	Skin burning sensation	26	-2.9	-3.5
	Anaesthesia	1	0.2	-3.5
	hypoesthesia eye	1	-0.3	-4.1
	Burning mouth syndrome	1	-0.3	-4.1
	Paraesthesia mucosal	1	-0.7	-4.5
	Pharyngeal hypoesthesia	1	-1.4	-5.2
	Burning sensation mucosal	1	-1.7	-5.5
	Anaesthesia oral	2	-3.5	-6.1
Liraglutide				
	Paraesthesia oral	35	-0.4	-0.9
	Burning sensation	99	-0.9	-1.2
	hypoesthesia oral	24	-0.7	-1.4
	hypoesthesia	172	-1.3	-1.5
	Paraesthesia	197	-1.3	-1.6
	Formication	10	-1.0	-2.0
	Pharyngeal hypoesthesia	2	-0.4	-3.0
	Skin burning sensation	22	-2.8	-3.5
	hypoesthesia eye	1	-0.1	-3.9
	Burning mouth syndrome	1	-0.1	-3.9
	Hyperaesthesia	4	-2.2	-3.9
	Genital hypoesthesia	1	-0.4	-4.2
	Dysesthesia	2	-2.2	-4.8
	Pharyngeal paraesthesia	1	-1.6	-5.4
Lixisenatide				
	Burning sensation	4	0.4	-1.4
	Paraesthesia	6	-0.4	-1.8
	hypoesthesia	4	-0.7	-2.4
	Skin burning sensation	2	-0.3	-2.9
	Formication	1	0.8	-3.0
	hypoesthesia oral	1	0.4	-3.4
	Paraesthesia oral	1	0.2	-3.6
Semaglutide				
	Hyperaesthesia	157	2.6	2.4
	Dysesthesia	62	2.1	1.8
	Skin burning sensation	229	0.2	0.0
	Burning sensation	247	0.1	-0.1
	Paraesthesia oral	58	0.0	-0.4
	hypoesthesia oral	43	-0.2	-0.7
	Paraesthesia	417	-0.6	-0.7
	hypoesthesia	250	-1.1	-1.2
	Pharyngeal paraesthesia	4	-0.3	-2.1
	Cold dysesthesia	1	1.3	-2.5
	Intranasal paraesthesia	1	1.2	-2.6
	Dental paraesthesia	1	1.0	-2.8
	Pharyngeal hypoesthesia	2	-0.7	-3.3
	Eye paraesthesia	1	0.5	-3.3
	Formication	6	-2.0	-3.4
	Anaesthesia	1	0.2	-3.6
	Genital paraesthesia	1	0.2	-3.6
	Burning sensation mucosal	2	-1.0	-3.6
	hypoesthesia eye	1	-0.3	-4.1
	Burning mouth syndrome	1	-0.3	-4.1
	Genital hypoesthesia	1	-0.6	-4.4
Tirzepatide				
	Hyperaesthesia	52	0.7	0.3
	Dysesthesia	24	0.4	-0.2
	Burning mouth syndrome	4	1.0	-0.8
	Paraesthesia oral	56	-0.4	-0.8
	hypoesthesia oral	42	-0.6	-1.1
	Paraesthesia	325	-1.3	-1.4
	Burning sensation	119	-1.3	-1.5
	hypoesthesia	211	-1.7	-1.9
	Skin burning sensation	77	-1.7	-2.1
	Anal hypoesthesia	1	1.2	-2.6
	Paraesthesia ear	1	0.4	-3.4
	Pharyngeal hypoesthesia	2	-1.0	-3.6
	Pharyngeal paraesthesia	2	-1.5	-4.1
	Formication	2	-3.7	-6.3
	Anaesthesia oral	1	-4.6	-8.4
