# Supplementary material for: Dysesthesia associated with GLP-1 agonist therapies: data-mining analysis and literature review
Source: Eur J Clin Pharmacol. 2026 May 22;82(6):154. doi: 10.1007/s00228-026-04079-7 (PMC13194321; doi:10.1007/s00228-026-04079-7)
Supplement: Supplementary file 1 — Supplementary Material 1. [file 228_2026_4079_MOESM1_ESM.docx]

**Dysesthesia associated with GLP-1 Agonist Therapies: data-mining analysis and literature review**

**Appendix 1: Information component (IC) and IC025 of individual cases of suspected adverse reactions to GLP-1R agonists associated with dysesthesia reported in VigiBase®**

| **Drug** | **Reaction (PT)** | **Number** | **IC** | **IC025** |
| --- | --- | --- | --- | --- |
| Albiglutide |  |  |  |  |
|  | Burning sensation | 12 | -0.9 | -1.8 |
|  | Hypoesthesia | 13 | -2.0 | -2.9 |
|  | Paraesthesia | 14 | -2.1 | -3.0 |
|  | Skin burning sensation | 5 | -1.9 | -3.4 |
|  | hypoesthesia oral | 2 | -1.2 | -3.8 |
|  | Paraesthesia oral | 1 | -2.1 | -5.9 |
| Dulaglutide |  |  |  |  |
|  | Burning sensation | 132 | -0.7 | -1.0 |
|  | hypoesthesia oral | 33 | -0.6 | -1.1 |
|  | Pharyngeal paraesthesia | 6 | 0.2 | -1.2 |
|  | hypoesthesia | 253 | -1.0 | -1.2 |
|  | Paraesthesia oral | 29 | -1.0 | -1.5 |
|  | Paraesthesia | 186 | -1.7 | -1.9 |
|  | Pharyngeal hypoesthesia | 2 | -0.7 | -3.3 |
|  | Hyperaesthesia | 6 | -1.9 | -3.3 |
|  | Anaesthesia | 1 | 0.3 | -3.5 |
|  | Formication | 5 | -2.2 | -3.7 |
|  | Skin burning sensation | 19 | -3.3 | -4.1 |
|  | Burning mouth syndrome | 1 | -0.3 | -4.1 |
|  | Dysesthesia | 1 | -3.2 | -7 |
| Exenatide |  |  |  |  |
|  | hypoesthesia oral | 89 | 0.8 | 0.5 |
|  | Paraesthesia oral | 79 | 0.5 | 0.1 |
|  | hypoesthesia | 376 | -0.5 | -0.6 |
|  | Burning sensation | 154 | -0.5 | -0.8 |
|  | Paraesthesia | 362 | -0.8 | -0.9 |
|  | Formication | 19 | -0.4 | -1.1 |
|  | Hyperaesthesia | 13 | -0.9 | -1.8 |
|  | Pharyngeal paraesthesia | 3 | -0.7 | -2.7 |
|  | Dysesthesia | 4 | -1.6 | -3.4 |
|  | Skin burning sensation | 26 | -2.9 | -3.5 |
|  | Anaesthesia | 1 | 0.2 | -3.5 |
|  | hypoesthesia eye | 1 | -0.3 | -4.1 |
|  | Burning mouth syndrome | 1 | -0.3 | -4.1 |
|  | Paraesthesia mucosal | 1 | -0.7 | -4.5 |
|  | Pharyngeal hypoesthesia | 1 | -1.4 | -5.2 |
|  | Burning sensation mucosal | 1 | -1.7 | -5.5 |
|  | Anaesthesia oral | 2 | -3.5 | -6.1 |
| Liraglutide |  |  |  |  |
|  | Paraesthesia oral | 35 | -0.4 | -0.9 |
|  | Burning sensation | 99 | -0.9 | -1.2 |
|  | hypoesthesia oral | 24 | -0.7 | -1.4 |
|  | hypoesthesia | 172 | -1.3 | -1.5 |
|  | Paraesthesia | 197 | -1.3 | -1.6 |
|  | Formication | 10 | -1.0 | -2.0 |
|  | Pharyngeal hypoesthesia | 2 | -0.4 | -3.0 |
|  | Skin burning sensation | 22 | -2.8 | -3.5 |
|  | hypoesthesia eye | 1 | -0.1 | -3.9 |
|  | Burning mouth syndrome | 1 | -0.1 | -3.9 |
|  | Hyperaesthesia | 4 | -2.2 | -3.9 |
|  | Genital hypoesthesia | 1 | -0.4 | -4.2 |
|  | Dysesthesia | 2 | -2.2 | -4.8 |
|  | Pharyngeal paraesthesia | 1 | -1.6 | -5.4 |
| Lixisenatide |  |  |  |  |
|  | Burning sensation | 4 | 0.4 | -1.4 |
|  | Paraesthesia | 6 | -0.4 | -1.8 |
|  | hypoesthesia | 4 | -0.7 | -2.4 |
|  | Skin burning sensation | 2 | -0.3 | -2.9 |
|  | Formication | 1 | 0.8 | -3.0 |
|  | hypoesthesia oral | 1 | 0.4 | -3.4 |
|  | Paraesthesia oral | 1 | 0.2 | -3.6 |
| **Semaglutide** |  |  |  |  |
|  | Hyperaesthesia | 157 | 2.6 | 2.4 |
|  | Dysesthesia | 62 | 2.1 | 1.8 |
|  | Skin burning sensation | 229 | 0.2 | 0.0 |
|  | Burning sensation | 247 | 0.1 | -0.1 |
|  | Paraesthesia oral | 58 | 0.0 | -0.4 |
|  | hypoesthesia oral | 43 | -0.2 | -0.7 |
|  | Paraesthesia | 417 | -0.6 | -0.7 |
|  | hypoesthesia | 250 | -1.1 | -1.2 |
|  | Pharyngeal paraesthesia | 4 | -0.3 | -2.1 |
|  | Cold dysesthesia | 1 | 1.3 | -2.5 |
|  | Intranasal paraesthesia | 1 | 1.2 | -2.6 |
|  | Dental paraesthesia | 1 | 1.0 | -2.8 |
|  | Pharyngeal hypoesthesia | 2 | -0.7 | -3.3 |
|  | Eye paraesthesia | 1 | 0.5 | -3.3 |
|  | Formication | 6 | -2.0 | -3.4 |
|  | Anaesthesia | 1 | 0.2 | -3.6 |
|  | Genital paraesthesia | 1 | 0.2 | -3.6 |
|  | Burning sensation mucosal | 2 | -1.0 | -3.6 |
|  | hypoesthesia eye | 1 | -0.3 | -4.1 |
|  | Burning mouth syndrome | 1 | -0.3 | -4.1 |
|  | Genital hypoesthesia | 1 | -0.6 | -4.4 |
| **Tirzepatide** |  |  |  |  |
|  | Hyperaesthesia | 52 | 0.7 | 0.3 |
|  | Dysesthesia | 24 | 0.4 | -0.2 |
|  | Burning mouth syndrome | 4 | 1.0 | -0.8 |
|  | Paraesthesia oral | 56 | -0.4 | -0.8 |
|  | hypoesthesia oral | 42 | -0.6 | -1.1 |
|  | Paraesthesia | 325 | -1.3 | -1.4 |
|  | Burning sensation | 119 | -1.3 | -1.5 |
|  | hypoesthesia | 211 | -1.7 | -1.9 |
|  | Skin burning sensation | 77 | -1.7 | -2.1 |
|  | Anal hypoesthesia | 1 | 1.2 | -2.6 |
|  | Paraesthesia ear | 1 | 0.4 | -3.4 |
|  | Pharyngeal hypoesthesia | 2 | -1.0 | -3.6 |
|  | Pharyngeal paraesthesia | 2 | -1.5 | -4.1 |
|  | Formication | 2 | -3.7 | -6.3 |
|  | Anaesthesia oral | 1 | -4.6 | -8.4 |
